# Association of Pain Widespreadness in Pain Drawings With Psychological Burden Among Tertiary Care Patients With Temporomandibular Disorders

**DOI:** 10.1155/ijod/6610627

**Published:** 2026-10-22

**Authors:** Arvid Iljin, Kirsi Sipilä, Tuija Suvinen, Tuija Teerijoki-Oksa, Ville Vuollo, Ritva Näpänkangas

**Affiliations:** ^1^ Research Unit of Population Health University of Oulu Oulu Finland oulu.fi; ^2^ Medical Research Center Oulu University Hospital University of Oulu Oulu Finland oulu.fi; ^3^ University of Turku Turku Finland utu.fi

**Keywords:** anxiety symptoms, DC/TMD, depression symptoms, pain drawing, psychosocial, widespread pain

## Abstract

**Objective:**

The aim was to assess the association between pain widespreadness and psychological burden among tertiary care temporomandibular disorders (TMDs) pain patients by utilizing pain drawings (PDs) of the diagnostic criteria for TMD (DC/TMD)‐FIN both visually and quantitatively.

**Methods:**

A total of 197 TMD pain patients were divided into PD profile subgroups (PD‐1 = head/face pain; PD‐2 = head and neck/shoulder regional pain; PD‐3 = widespread pain). In quantitative analysis of PDs, PD score was calculated, considering the pain widespreadness. Subscales and median sum scores of generalized anxiety disorder‐7 (GAD‐7) and patient health questionnaires (PHQ‐9 and PHQ‐15) were calculated. Differences in subscales and median sum scores of GAD‐7, PHQ‐9, and PHQ‐15 between PD profile subgroups were evaluated with independent samples Kruskal–Wallis and chi‐square tests. Linear regression models for PD sum score included sum scores of GAD‐7, PHQ‐9, and PHQ‐15, sociodemographic variables, pain duration, and study site. When using PD profile subgroup as outcome ordinal logistic regression models were used.

**Results:**

Visualized via PDs, mean sum scores of GAD‐7, PHQ‐9, and PHQ‐15 increased significantly from PD‐1 to PD‐3 profile subgroup. Based on quantitative analysis, mean PD sum scores were highest among patients with severe anxiety, depression, and highest somatic symptom levels. PD sum scores tended to increase with anxiety and depression severity, but statistically significant subgroup differences were observed only for PHQ‐15. In the multivariate models, sum scores of GAD‐7, PHQ‐9, and PHQ‐15 were significantly associated with higher PD scores and higher PD profile subgroup.

**Conclusion:**

Utilizing PD as an assessment tool both visually and quantitatively, patients with widespread pain profiles demonstrated higher levels of anxiety, depression, and somatic symptoms. Widespread pain may refer to higher psychosocial burden in TMD pain patients.

## 1. Introduction

Temporomandibular disorders (TMDs) and pain are conditions affecting the masticatory muscles, temporomandibular joints (TMJs), and related areas [1, 2]. These disorders share characteristics with general musculoskeletal and chronic pain conditions, whose background is based on a biopsychosocial model [3, 4]. This model considers not only the physical aspects of illness but also the biopsychosocial impact factors on health [3]. Particularly in cases of chronic pain, a broader perspective that includes psychological, behavioral, and social factors, as well as overall well‐being, should be evaluated [2–8].

Patients suffering from TMD pain frequently exhibit psychological comorbidities such as anxiety and depression, along with nonspecific physical symptoms [9–13]. Studies have shown that individuals with TMD pain who report a heavier biopsychosocial burden tend to report more comorbid and widespread pain [11–14]. Furthermore, those with more severe TMD pain‐related psychosocial disability often have increased psychological distress, including symptoms of depression and anxiety, compared to those with less disability [15, 16].

Patients with TMD pain often report multiple pain sites in various parts of the body, including the back, stomach, legs, hands, as well as headaches and fibromyalgia [13, 14]. The psychological profiles of patients are known to influence their pain perception [17] and can lead to the development of prolonged, chronic, and widespread pain [13]. Conversely, enduring widespread TMD pain may also contribute to depressive mood or anxiety [18, 19]. This bidirectional relationship highlights the complex interplay between psychological factors and the pain experience in TMD patients.

The diagnostic criteria for TMDs (DC/TMD) include Axis I for assessing TMD clinical diagnoses and Axis II for assessing pain‐related impact and psychological and psychosocial risk factors as well as whole‐body pain site distribution [2]. Pain drawing (PD) has been added as a new instrument in the DC/TMD criteria for visualizing and assessing pain locations, distribution, and widespreadness of TMD pain and comorbid pain problems [2, 20]. Studies concerning the use of PDs in TMD pain patients are scarce [1, 20–22]. Previous studies have used traditional methods, that is, analyzing PDs with paper and pencil and transparent templates [13, 21]. Also, computerized methods of analyzing PDs have also been presented in different studies on pain patients [23–28]. In our previous study [29], using PD profiling of TMD pain patients, those with widespread pain profiles reported higher pain‐related interference/disability and more self‐reported comorbid pain problems and pain‐related Axis I diagnoses. However, more studies are needed to evaluate the applicability of PDs in TMD pain patient assessments in the context of other biopsychosocial assessments and pain patient profiling.

The aim of the study was to assess the association between pain widespreadness and psychological burden among tertiary care TMD pain patients by utilizing the PDs of the DC/TMD‐FIN both visually and quantitatively. It can be hypothesized that pain widespreadness, assessed through quantitative analysis of PDs within the DC/TMD‐FIN framework, is significantly associated with increased psychological burden among tertiary care TMD pain patients.

## 2. Materials and Methods

A total of 197 TMD pain patients were consecutively included in the study based on referral for TMD treatment to tertiary care facial pain clinics in Helsinki (*n* = 125), Kuopio (*n* = 19), Oulu (*n* = 9), and Turku (*n* = 44) University Hospitals between July 2015 and March 2019. All patients with clinically diagnosed TMD and aged 17 years or older were included. The age range of the patients was 17–83 years (mean age 43.3 years), and there were 158 women and 39 men. Participation was voluntary, and the subjects provided written consent. The Ethics Committee of the Hospital District of Southwest Finland has approved the study (74/1082/2015).

### 2.1. DC/TMD PD

The clinical TMD examinations were performed by four calibrated and reliability‐trained examiners according to the Finnish version of DC/TMD (DC/TMD‐FIN) [2, 30, 31]. Before the TMD examination visit, the patients completed the PD of the DC/TMD‐FIN on a standard A4 sheet at home, along with other DC/TMD Axis II questionnaires. They were instructed to shade all the areas on their body and face/head where they currently felt pain, indicating each specific pain site. The PDs were evaluated for accuracy by the treating clinician at the visit.

The PDs were independently analyzed (by author Arvid Iljin, who was not involved in the clinical examination and was unaware of the other patient data) by using a transparent assessment template, the details of the method are described in a previous publication [29]. Based on PDs, patients were classified in three PD profile subgroups (PD‐1–3) [16]: PD‐1 = local pain, pain located only in the facial and head area; PD‐2 = regional pain, pain located in the trigeminal‐cervical region (including head and neck/shoulder regions); and PD‐3 = widespread pain, local and/or regional and multiple bodily pain sites outside the areas of PD‐1 and PD‐2) (Figure 1a). In total, 196 TMD pain patients completed PD assessments. Each patient was classified only into one PD profile subgroup. The total number of patients in the PD‐1 profile subgroup was 60 (30.6%), in PD‐2 74 patients (37.8%), and in PD‐3 subgroup 62 patients (31.6%) [29].

**Figure 1:**
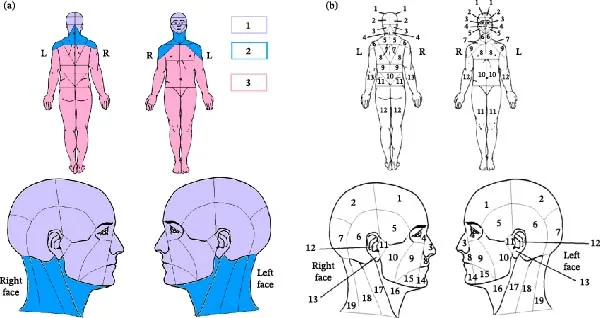
(a)DC/TMD pain drawing (PD) divided into three PD profile subgroups (PD 1–3; PD‐1 (purple): local pain, pain located only in facial and head area, PD‐2 (blue): regional pain pain located in the trigeminal‐cervical region (including head and neck/shoulder regions), PD‐3 (pink): widespread pain, local and/or regional and multiple bodily pain sites outside the areas of PD‐1 and PD‐2. (b) DC/TMD pain drawings divided into smaller sections for quantitative analysis by calculating means of sections within PD‐1 (local pain, pain located only in facial and head area, facial area sections 1–19), PD‐2 (regional pain, pain located in the trigeminal‐cervical region (including head and neck/shoulder regions; facial area sections 1–19 and whole body sections 1–7 anterior and 1–6 posterior view), PD‐3 (widespread pain, local and/or regional and multiple bodily pain sites outside the areas of PD‐1 and PD‐2, facial area sections 1–19 and whole body sections 1–12 anterior and 1–13 posterior view.

### 2.2. Quantitative Analysis of DC/TMD PDs

For more detailed quantitative analysis, the PDs were divided into smaller sections by PD profile subgroups: PD‐1: facial area, PD‐2: facial and neck area, and PD‐3: facial and neck areas and whole‐body pain (Figure 1b) [29]. The PD score was defined from 0 to 4 points depending on the size of the area that was marked as a pain area (0 = 0%, 1 point = 1%–33%, 2 points = 34%–66%, 3 points = 67%–99%, and 4 points = 100% of the section was marked as a pain area). All PD sections/areas were analyzed and assessed by using a transparent sheet independently three times.

### 2.3. Anxiety, Depression, and Physical Symptoms

Using the DC/TMD‐FIN Axis II questionnaires, the patients reported how much they had suffered from anxiety symptoms (generalized anxiety disorder‐7 [GAD‐7]‐FIN), depression symptoms (patient health questionnaire [PHQ]‐9‐FIN), and physical symptoms (PHQ‐15‐FIN).

The sum scores of each questionnaire were calculated, and they were further classified based on DC/TMD Axis II scoring criteria as follows: GAD‐7 (mild, moderate, and severe), PHQ‐9 (mild, moderate, moderate‐severe, and severe), and PHQ‐15 (low, medium, and high) [2, 20, 32, 33].

• Anxiety (GAD‐7‐FIN, seven questions, range 0–3, where 0 is “not at all” and 3 “nearly every day”) during the last 2 weeks; sum score classified as: ≥5 mild anxiety, ≥10 moderate anxiety, ≥15 severe anxiety.
• Depression (PHQ‐9‐FIN, nine questions, range 0–3, where 0 is “not at all” and 3 “nearly every day”) during the past 2 weeks; sum score classified as: ≥5 mild depression, ≥10 moderate depression, ≥15 moderate‐severe depression, and ≥ 20 severe depression
• Physical symptoms (PHQ‐15‐FIN, 15 questions, range 0–2, where 0 is “not bothered” and 2 “bothered a lot during the past 4 weeks”), sum score classified as ≥5 low symptom severity, ≥10 medium symptom severity, ≥15 high symptom severity.

In sum scores of GAD‐7, PHQ‐9, and PHQ‐15 questionnaires, missing values were replaced with the mean value of other items. If there were missing values for more items than the following limits, the response was considered as missing for the instrument: GAD‐7: two items (*n* = 6), PHQ‐9: three items (*n* = 4), and PHQ‐15: five items (*n* = 4) [32].

The number of available data from these questionnaires were 191, 192, and 193 for GAD‐7, PHQ‐9, and PHQ‐15, respectively.

### 2.4. Sociodemographic Data

Sociodemographic data was gathered using the DC/TMD‐FIN demographics questionnaire [20]. The number of responses were as follows: age (*n* = 196), marital status (*n* = 192), education level (*n* = 193), and employment status (*n* = 193). For analysis, marital status, educational level, and employment status were reclassified for analyses according to Iljin et al. [29]. The duration of facial pain was inquired with the following question: “How many years ago was the onset of pain in facial area”? (188 responses)

### 2.5. Statistical Analysis

The distribution of PD profile subgroups was described by frequencies and percentages. In the primary analyses, associations of PD profile subgroups with GAD‐7, PHQ‐9, and PHQ‐15 subscales were evaluated by using chi‐square tests. The differences in GAD‐7, PHQ‐9, and PHQ‐15 sum scores between PD profile subgroups were evaluated with independent samples Kruskal–Wallis tests. The statistical significance was set at *p*  < 0.05. In pairwise comparisons of GAD‐7, PHQ‐9, and PHQ‐15 sum scores (differences between PD‐3 vs. 1 and 2; and PD‐2 vs. 1 and 3) Dunn’s test was used.

The mean PD score for each section was calculated and color‐coded. The sum of PD scores (0–4) was calculated and compared between GAD‐7, PHQ‐9, and PHQ‐15 subscales by using independent samples Kruskal–Wallis tests. Pairwise comparisons were performed using the Dunn’s test.

In the secondary analyses, the associations of PD profile subgroups with gender, marital status, education level, and working status were evaluated with the chi‐square test. Distribution of sociodemographic variables was also compared between study sites using the chi‐square test. The associations of PD profile subgroups with age and duration of facial pain were evaluated with independent sample Kruskal–Wallis tests. The associations of these background factors with GAD‐7, PHQ‐9, and PHQ‐15 were evaluated using the chi‐square test (for classified variables) and independent samples Kruskal–Wallis tests (for continuous variables).

Linear regression analyses were performed using the PD sum score as a dependent variable and including sum core of GAD‐7, PHQ‐9, and PHQ‐15 (separately), gender, age, education level, pain duration per year, and study site as independent variables. Also, ordinal logistic regression analysis (using PD profile subgroup as outcome variable) was used to evaluate using the same confounders.

Data were analyzed by using SPSS for Windows version 29 (SPSS Inc., Chicago, Ill., USA).

## 3. Results

The associations between PD profile subgroups and sociodemographic variables are presented in Table 1. The gender distribution differed significantly between the PD profile subgroups. The proportion of females was lower in PD‐1 as compared to that in PD‐2 and PD‐3 subgroups. The other sociodemographic variables did not significantly differ between PD profiles. The study pain sites did not significantly differ with regard to sociodemographic factors or pain duration (Table S1).

**Table 1:** Association of PD profiles with sociodemographic factors and pain duration among TMD pain patients in tertiary care (*n* = 197).

Variable	PD‐1 (*n* = 60)	PD‐2 (*n* = 74)	PD‐3 (*n* = 62)	*p*
Females	41 (68.3%)	64 (86.5%)	52 (83.9%)	0.022^a^
Age (mean, SD)	40.6 (17.1)	42.5 (16.5)	46.7 (16.6)	0.098^b^
Pain duration/yrs (mean, SD)	7.0 (10.7)	12.2 (35.5)	5.5 (6.2)	0.667^b^
Marital status/single	19 (32.2%)	33 (45.2%)	43 (29.5%)	0.126^a^
Lower education	40 (67.8%)	37 (50.7%)	39 (65.0%)	0.092^a^
Unemployed	26 (44.8%)	35 (47.9%)	24 (60.7%)	0.180^a^

^a^Chi‐square test.

^b^Kruskal–Wallis test.

Distribution of GAD‐7, PHQ‐9, and PHQ‐15 subscales by PD 1–3 subgroups is presented in Table 2. The PD subgroup had no significant associations with the GAD‐7 or PHQ‐9 subscale (Table 2). PD‐subgroups associated significantly with PHQ‐15 subscale (*p*  < 0.001). TMD pain patients in the PD‐3 profile subgroup had the highest proportion (46.7%) of the high subscale of PHQ‐15, while the corresponding proportions of the PD‐3 subgroup in other PHQ‐15 subscales were much lower.

**Table 2:** Distribution (frequencies and percentages) of subscales and sum scores (medians and interquartile ranges, IQR) of GAD‐7, PHQ‐9, and PHQ‐15 in pain drawing (PD) 1–3 subgroups among TMD pain patients in tertiary care (*n* = 197).

PD‐subgroup	GAD‐7 (anxiety symptoms)						
	No	Mild	Moderate	Moderate‐ severe/severe	*p* ^a^	Median (IQR)	*p* ^b^
PD‐1	43 (74.1%)	9 (15.5%)	4 (6.9%)	2 (3.4%)	0.072	3.0 (1.0–5.3)	0.041
PD‐2	40 (54.8%)	23 (31.5%)	6 (8.2%)	4 (5.5%)		3.0 (1.0–7.0)	
PD‐3	28 (47.5%)	17 (28.8%)	7 (11.9%)	7 (11.9%)		5.0 (2.0–9.0)	

PD‐subgroup	PHQ‐9 (depression symptoms)					
	No/mild	Moderate	Moderate–severe/severe	*p* ^a^	Median (IQR)	*p* ^b^
PD‐1	53 (91.4%)	2 (3.4%)	3 (5.2%)	0.097	3.0 (0.5–5.5)	<0.001
PD‐2	57 (78.1%)	10 (13.7%)	6 (8.2%)		4.0 (1.0–9.0)	
PD‐3	44 (72.1%)	11 (18.0%)	6 (9.8%)		6.8 (3.0–10.0)	

PD‐subgroup	PHQ‐15 (physical symptoms)						
	No	Low	Medium	High	*p* ^a^	Median (IQR)	*p* ^b^
PD‐1	15 (25.4%)	25 (42.4%)	15 (25.4%)	4 (6.8%)	<0.001	7.0 (4.3–10.7)	<0.001
PD‐2	9 (12.3%)	37 (50.7%)	18 (24.7%)	9 (12.3%)		8.1 (6.0–11.0)	
PD‐3	1 (1.7%)	15 (25.0%)	16 (26.7%)	28 (46.7%)		14.0 (9.2–17.8)	

^a^Chi‐square ‐test.

^b^Independent samples Kruskal–Wallis test.

Median sum scores of GAD‐7, PHQ‐9, and PHQ‐15 increased from PD‐1 to PD‐3 subgroup, differences being statistically significant in all variables (Table 2). In pairwise comparisons after Bonferroni corrections, sum scores of both GAD‐7 and PHQ‐9 showed significant differences between PD‐1 and PD‐3 profile subgroups (*p* = 0.040 and *p*  < 0.001, respectively). Sum score of PHQ‐15 showed significant differences between PD‐1 and PD‐3 subgroups (*p* < 0.001) and between PD‐2 and PD‐3 subgroups (*p* < 0.001).

Based on quantitative analysis, median PD sum scores associated significantly with subscales of GAD‐7, PHQ‐9, and PHQ‐15, that is, the median PD sum scores were highest among those patients with the highest subscale of anxiety, depression, and physical symptoms (Table 3). Pairwise comparisons after Bonferroni corrections showed significant associations between the PHQ‐15 subscales.

**Table 3:** Pain drawing (PD) sum scores (medians and interquartile ranges, IQR) in PHQ‐9, PHQ‐15, and GAD‐7 subscales.

Variable	*n*	Median (IQR)	*p* ^a^	Pairwise comparisons^b^
GAD‐7				
1. No	111	1.8 (0.8–3.1)	0.051	ns.
2. Mild	49	2.4 (1.4–4.5)		
3. Moderate	17	2.8 (1.6–4.8)		
4. Moderate/severe	13	3.7 (0.9–5.3)		
PHQ‐9				
1. No/mild	154	1.6 (0.7–2.8)	0.078	ns.
2. moderate	23	3.0 (1.4–4.8)		
3. Moderate–severe/severe	15	2.4 (0.7–3.9)		
PHQ‐15				
1. No	25	0.9 (0.4–1.9)	<0.001	1 vs. 3: *p* = 0.045 1 vs. 4: *p* < 0.001 2 vs. 4: *p* = 0.036
2. Low	77	2.0 (1.2–3.4)	—	
3. Medium	49	2.4 (1.0–3.8)	—	
4. High	41	3.8 (2.0–5.4)	—	

^a^Independent samples Kruskal–Wallis test.

^b^Dunn’s test.

In the linear regression analyses (using PD score as outcome variable), sum scores of GAD‐7, PHQ‐9, and PHQ‐15 were significantly associated with higher PD scores (Table 4). No statistically significant associations were observed for gender, age, pain duration, education, or study site. In the ordinal logistic regression analysis (using PD profile subgroup as outcome variable), gender, age, GAD‐7 sum core, and study site were significantly associated with PD profile subgroup, whereas educational level and pain score were not significant predictors (Model 1, Table 4). In Models 2 and 3, PHQ‐9 and PHQ‐15 sum scores, as well as age and gender, were significantly associated with the PD profile subgroup.

**Table 4:** Pain drawing sum score (using linear regression models) and PD profile subgroup (using ordinal logistic regression model) as outcome variables, including GAD‐7, PHQ‐9, and PHQ‐15 (in separate models), gender, age, education level, study site, and pain duration (per years) as explanatory variables.

Variable	Outcome: PD sum score				Outcome: PD profile subgroups			
	95% CI				95% CI			
	Beta^a^	Lower	Upper	*p*	Estimate (*β*)	Lower	Upper	*p*
Model 1 (*n* = 138)								
GAD‐7	0.104	0.039	0.169	0.002	0.090	0.030	0.150	0.003
Gender	0.529	−0.250	1.307	0.181	0.986	0.268	10.704	0.007
Age	0.002	−0.017	0.022	0.821	0.023	0.005	0.040	0.010
Education	−0.010	−0.661	0.642	0.977	0.108	−0.478	0.694	0.718
Study site	0.273	−0.062	0.609	0.109	0.418	0.102	0.734	0.010
Pain duration	−0.017	−0.051	0.017	0.334	−0.017	−0.049	0.014	0.281
Model 2 (*n* = 140)								
PHQ‐9	0.123	0.062	0.184	<0.001	0.113	0.053	0.173	<0.001
Gender	0.624	−0.131	1.378	0.104	0.986	0.268	10.704	0.007
Age	0.002	−0.017	0.021	0.833	0.023	0.004	0.038	0.019
Education	−0.121	−0.757	0.515	0.708	−0.013	−0.600	0.574	0.965
Study site	0.160	−0.167	0.488	0.335	0.302	−0.014	0.617	0.061
Pain duration	−0.014	−0.047	0.020	0.421	−0.015	−0.047	0.017	0.350
Model 3 (*n* = 141)								
PHQ‐15	0.178	0.126	0.229	<0.001	0.212	0.146	0.277	<0.001
Gender	0.324	−0.361	1.008	0.351	0.800	0.061	10.538	0.034
Age	−0.003	−0.020	0.014	0.737	0.019	0.001	0.037	0.036
Education	0.286	−0.294	0.867	0.330	0.397	−0.215	10.009	0.203
Study site	0.200	−0.097	0.497	0.185	0.364	0.036	0.693	0.030
Pain duration	−0.019	−0.049	0.012	0.230	−0.023	−0.055	0.010	0.173

^a^Unstandardized coefficient.

## 4. Discussion

In the present study, TMD pain patients were categorized visually into PD‐1–PD‐3 subgroups and analyzed also quantitatively by PD scores based on the extent of pain markings in PDs. These subgroups served as indicators of widespread pain. The results showed that pain widespreadness associated significantly with the severity of anxiety, depressive, and physical symptoms (based on sum score variables) among Finnish tertiary care TMD pain patients, showing that those with widespread pain had significantly more severe psychological symptoms than those with more localized pain. The associations were clearer when symptom burden was handled as a continuous variable, whereas categorical severity levels did not uniformly differ. The medians for sums for PHQ‐9 and PHQ‐15 were approximately two‐fold in PD‐3 subgroups as compared to the PD‐1 subgroup. The PD‐2 subgroup acted as an intermediate group concerning the psychological burden. In the multivariate models, the associations of psychological measures were significant when using both PD sum score and PD profile subgroup as outcome variable. These results indicate that the associations may be clinically meaningful.

The most significant variable associating with widespread pain was the presence of nonspecific physical symptoms/somatization. The PHQ‐15 instrument reflects somatic symptom burden, which refers to a phenomenon in which psychological distress manifests as physical symptoms, such as pain, fatigue, or other bodily sensations. It is often associated with situations where no clear organic explanation for the symptoms can be found or where psychological factors clearly worsen the symptoms. In pain patients, this may appear as intensification of pain during periods of stress and anxiety.

The present results support the previous findings [34, 35]. In a study by Jo et al. [35] in a sample of young female TMD patients (*n* = 45), higher somatization was found among those with widespread pain as compared to those with localized pain. They also showed that conventional TMD treatment reduced pain intensity less in the widespread pain group as compared to the localized pain group, thus indicating that the presence of widespread pain should be identified and considered in the treatment of TMD. Additionally, a population‐based follow‐up study showed that somatization predicts chronic widespread pain [36]. Further, a Chinese case–control study (200 participants with orofacial pain vs. 200 pain‐free controls) showed that comorbid widespread pain occurred relatively often, and psychological distress was common in patients with oral facial pain, particularly in those with widespread pain [34].

In this study, TMD pain patients with moderate to severe anxiety levels showed a tendency for wider pain in the head, neck, and shoulder area than those with no or low levels of anxiety. Anxiety involves worry, fear, misery, and psychological stress experienced by the person, and anxiety symptoms have been shown to exist especially among patients with muscle‐related TMD diagnoses [37]. In a study by Ji et al. [38], TMD pain patients with widespread pain showed higher levels of anxiety than those with local pain, thus supporting the present findings. It has also been argued that anxiety can increase the amount of parafunction [39, 40].

The association of widespread pain with psychological symptoms may be explained by central sensitization, which has been found to be related to TMD [41] and chronic pain [38]. Based on a systematic review, in premorbid and acute stage high sensory sensitivity and/or somatization were the strongest predictors of altered central pain modulation in chronic musculoskeletal pain [42]. Further, it has been shown that increased depressive mood accelerates ventromedial prefrontal cortex function, increasing pain sensitivity, anxiety, and distress levels [43]. On the other hand, it has to be taken into account that widespread pain experienced by TMD pain patients can result in increase of distress symptoms such as depressive mood as well as appearance and severeness of anxiety and physical symptoms [18, 19]. In addition, chronic pain, defined as pain lasting over 3 months, involves neuroplastic changes in the central nervous system that lower the pain threshold and may lead to more widespread pain and intensified physical symptoms [44–47].

Only a few studies exist on PDs and psychological instruments among TMD pain patients. A study by Suvinen et al. [13] from 135 TMD pain patients, using a similar method based on PDs as in the present study, reported that those with widespread pain had significantly higher depressive mood and pain somatization levels compared to TMD pain patients with localized pain [13]. It is; however, noteworthy that in the present study, DC/TMD Axis II instruments assessing depression used a shorter timeline (2 weeks) than RDC/TMD instruments (1 month) used in the earlier study by Suvinen et al. [13], which may have influenced some of the reported differences between PD profiling and depressive symptom scoring results (DC/PHQ‐9 assessments vs RDC/SCL‐90R assessments). Therefore, more psychometric studies related to, for example, DC/TMD cut‐off scoring are recommended [48]. Nevertheless, in another study by Pitance et al. [28], where digital DC/TMD PDs of 40 TMD pain patients were analyzed, showed also significantly wider body pain distribution among those patients with higher levels of pain intensity, anxiety levels, and central sensitivity than among patients with lower distress levels. In a study by Lövgren et al. [49], 300 individuals were recruited based on screening questions of TMD and examined according to the DC/TMD. Using PDs, they found that widespread pain was significantly associated with myofascial pain [49].

The PDs are important instruments for visual assessment of pain site localization in the facial/head and whole‐body areas, as also stated earlier in the study by Suvinen et al. [13]. The quantitative method of analysis of PDs takes into account not only the pain site locations but also the amount of the shaded area in each pain area drawn by the TMD pain patient [29]. In the present study, it was found that patients with severe anxiety, depression, and physical symptoms marked pain areas as more intense and wider than those with milder symptoms.

The strength of the present study is using the validated Finnish version of the DC/TMD in a large sample of TMD pain patients in a tertiary care setting. The sample size was originally determined based on the guidelines of the INFORM. Therefore, no a‐priori power analysis was made. This study evaluated additional variables that were not included in the original DC/TMD instrument scoring, such as classifications and scoring of PDs. The lack of power, which may lead to potential Type I or Type II errors, should be considered as a limitation of the study.

Based on PDs, patients were categorized into three distinct PD subgroups, enabling comparison of the psychological burden across varying levels of pain widespreadness. A novel quantitative method for interpreting PDs was applied, with all PDs analyzed independently by a single examiner who was blinded to other data of the patients [29]. However, this method could not take into account the pen used or the intensity of the marking, which may have affected the results. To improve reproducibility in future applications, the required marking tool (e.g., only B or 2B graphite pencils) should be specified. It should be noted that the PD subgroup and PD score are not fully independent analytical approaches as both of them indicate body pain widespreadness. The PD score also considers the pain extent in the distinct areas, whereas the PD subgroup describes more roughly the localization of body pain. The instruments for psychological symptoms were used both as classified and continuous. A sum score generally provides a more precise and informative estimate of the severity of depressive symptoms than a categorized variable because it preserves more variability and measures the amount or intensity of symptoms on a continuous scale. A weakness of the study was the lack of comparisons between genders due to the small amount of men in this study sample. It should also be noted that the use of mean substitution for missing questionnaire items may introduce bias. The division between low (no and mild) and high (moderate to severe) levels of anxiety, depressive mood, and physical symptom severity was chosen because of a small amount of TMD pain patients with some severe subclasses in this study. In addition, the cross‐sectional study does not resolve any causalities between pain widespreadness and psychological symptoms. Psychological factors and pain influence each other through a bidirectional relationship. The psychological burden can increase pain sensitization and weaken the pain‐inhibition mechanisms. At the same time, persistent pain increases the psychological burden, which further amplifies pain.

This study suggests that PDs can be used as a helpful visual and quantitative tool within the DC/TMD Axis II criteria. PDs help the clinicians to analyze comorbid pains and pain widespreadness, which also have a biopsychosocial impact in TMD pain patients. From the patient’s perspective, identifying a higher pain‐drawing profile may help ensure that care is better tailored to their overall symptom burden and daily challenges. Identification will support more individualized treatment planning, closer follow‐up, and timely referral to appropriate multidisciplinary services, potentially improving patient outcomes and reducing the risk of persistent disability and better quality of life.

In future studies, it would be interesting to evaluate the application of artificial intelligence to achieve more accurate analysis of PDs, which could potentially be applied also in clinical use. In addition, adopting a longitudinal study design to evaluate changes in pain and psychological symptoms over time would offer insights into the treatment outcomes of TMD.

## 5. Conclusions

When utilizing PD as an assessment tool both visually and quantitatively, patients with widespread pain profiles demonstrated higher levels of anxiety, depression, and somatic symptoms. Widespread pain may refer to a higher psychosocial burden in TMD pain patients.

## Funding

This study was supported by the Finnish Dental Society Apollonia.

## Conflicts of Interest

The authors declare no conflicts of interest.

## Supporting Information

Additional supporting information can be found online in the Supporting Information section.

## Supporting information


**Supporting Information** Table S1: Distribution of sociodemographic factors and pain duration by study unit among TMD pain patients in tertiary care (*n* = 197).

## Data Availability

The data that support the findings of this study are not publicly available due to the privacy or ethical restrictions.
