# Supplementary material for: Association of Pain Widespreadness in Pain Drawings With Psychological Burden Among Tertiary Care Patients With Temporomandibular Disorders
Source: Int J Dent. 2026 Oct 22;2026:6610627. doi: 10.1155/ijod/6610627 (PMC13691288; doi:10.1155/ijod/6610627)
Supplement: Supplementary file 1 [file IJOD-2026-6610627-s001.docx]

Table supplementary. Distribution of sociodemographic factors and pain duration by study unit

among TMD pain patients in tertiary care (n=197).

|  | 1 | 2 | 3 | 4 | P |
| --- | --- | --- | --- | --- | --- |
| Females | 8 (88.9%) | 100 (80.0%) | 13 (68.4%) | 37 (62.1%) | 0.473^†^ |
| Age (mean, SD) | 39.2 (18.4) | 42.4 (17.7) | 44.5 (16.9) | 43.2 (14.0) | 0.297^††^ |
| Pain duration/yrs (mean, SD) | 6.4 (5.7) | 6.5 (8.2) | 6.2 (9.3) | 8.6 (11.5) | 0.604^††^ |
| Lower education | 7 (77.8%) | 69 (56.6%) | 13 (68.4%) | 27 (62.8%) | 0.477^†^ |
| Unemployed | 3 (33.3%) | 66 (53.7%) | 8 (42.1%) | 21 (50.0%) | 0.555^†^ |

1=Kuopio University Hospital, 2= Helsinki University Hospital

3= Oulu University Hospital, 4= Turku University Hospital

^†^ Chi-square test ^††^ Kruskal-Wallis test
